# Characterization of tau prion seeding activity and strains from formaldehyde-fixed tissue

**DOI:** 10.1186/s40478-017-0442-8

**Published:** 2017-06-07

**Authors:** Sarah K. Kaufman, Talitha L. Thomas, Kelly Del Tredici, Heiko Braak, Marc I. Diamond

**Affiliations:** 1 Center for Alzheimer’s and Neurodegenerative Diseases, Peter O’Donnell Jr. Brain Institute, University of Texas Southwestern Medical Center, Dallas, TX USA; 2 Graduate Program in Neuroscience, Washington University in St. Louis, St. Louis, MO USA; 3 Clinical Neuroanatomy/Department of Neurology, Center for Biomedical Research, University of Ulm, Ulm, Germany

## Abstract

Tauopathies such as Alzheimer’s disease (AD) feature progressive intraneuronal deposition of aggregated tau protein. The cause is unknown, but in experimental systems trans-cellular propagation of tau pathology resembles prion pathogenesis. Tau aggregate inoculation into mice produces transmissible pathology, and tau forms distinct strains, i.e. conformers that faithfully replicate and create predictable patterns of pathology in vivo. The prion model predicts that tau seed formation will anticipate neurofibrillary tau pathology. To test this idea requires simultaneous assessment of seed titer and immunohistochemistry (IHC) of brain tissue, but it is unknown whether tau seed titer can be determined in formaldehyde-fixed tissue. We have previously created a cellular biosensor system that uses flow cytometry to quantify induced tau aggregation and thus determine seed titer. In unfixed tissue from PS19 tauopathy mice that express 1 N,4R tau (P301S), we have measured tau seeding activity that precedes the first observable histopathology by many months. Additionally, in fresh frozen tissue from human AD subjects at early to mid-neurofibrillary tangle stages (NFT I-IV), we have observed tau seeding activity in cortical regions predicted to lack neurofibrillary pathology. However, we could not directly compare the same regions by IHC and seeding activity in either case. We now describe a protocol to extract and measure tau seeding activity from small volumes (.04 mm^3^) of formaldehyde-fixed tissue immediately adjacent to that used for IHC. We validated this method with the PS19 transgenic mouse model, and easily observed seeding well before the development of phospho-tau pathology. We also accurately isolated two tau strains, DS9 and DS10, from fixed brain tissues in mice. Finally, we have observed robust seeding activity in fixed AD brain, but not controls. The successful coupling of classical IHC with seeding and strain detection should enable detailed study of banked brain tissue in AD and other tauopathies.

**Electronic supplementary material:**

The online version of this article (doi:10.1186/s40478-017-0442-8) contains supplementary material, which is available to authorized users.

## Introduction

Tauopathies are diverse neurodegenerative diseases characterized by the deposition of aggregated tau protein and progressive neuronal loss [18]. Each tauopathy has unique patterns of neuropathology, rates of progression, and regional involvement. This variability is reminiscent of distinct prionopathies, which are caused by prion protein (PrP) strains. Strains are unique aggregate structures that faithfully self-replicate, and produce distinct patterns of neuropathology [8, 20]. Tau resembles a PrP prion in experimental systems, as it mediates transmissible pathology in cells and animals, and transmits distinct disease pathologies by forming strains. Consequently, we use the term “prion” to describe tau, recognizing that there is no evidence that tau aggregates spontaneously transmit disease between individuals. We propose that a prion is best understood as a self-replicating assembly of defined structure that produces a specific biological effect, whether for good or ill. This definition encompasses an enormous literature on functional yeast prions and other mammalian proteins that utilize induced, self-amplifying conformational change to functional ends [23].

Strong evidence indicates that, like PrP, tau forms self-replicating strains that create unique patterns of pathology in cell and animal models [2, 7, 16, 22]. According to the prion model, uniquely structured tau assemblies form in one brain region, where they escape from affected cells. These “seeds” act as functional templates to trigger further protein aggregation following internalization by adjacent or synaptically connected cells.

It is still unknown whether this mechanism can account for progressive pathology in humans. A central challenge has been to determine the relationship of tau prion titer and strain composition to classical neuropathological descriptions of phospho-tau accumulation, which have been the gold standard for disease staging and discrimination among tauopathies [1, 15, 17]. Measurement of tau seeding activity in brain tissue and determination of strain composition will facilitate characterization of neuropathological specimens, and may help answer this question.

We have previously engineered HEK293T cells to detect tau seeding activity in unfixed tissues [13] and to isolate and characterize tau prion strains present in human brain [22]. In transgenic mouse models, the seed biosensor assay detects the emergence of pathological tau prions far in advance of frank neuropathology [13]. In selected fresh frozen brain samples from Alzheimer’s disease (AD) patients, these methods indicate that seeding activity might predict the accumulation of phospho-tau in characteristic neurofibrillary tangles (NFTs) [11]. However, because it has been impossible to directly compare adjacent, thin sections of brain, we have been unable to use the biosensor assay to simultaneously compare tau pathology with microscopy, seeding activity, or strain composition. We now describe assessment of tau seeding activity and strain composition in small amounts of fixed brain tissue from mice and humans.

## Methods

### Cell culture and cell lines

Seeding assay experiments were performed with a previously published biosensor cell line that expresses a fusion between 4R tau repeat domain (RD) containing the disease-associated P301S mutation, and cyan or yellow fluorescent proteins (tauRD(P301S)-CFP/YFP). These cells are freely available (ATCC CRL-3275) [13]. LM1, DS1, DS9, and DS10 are monoclonal HEK cell lines that were previously described [22]. They are derived from a monoclonal HEK cell line that stably expresses tauRD(P301L/V337M)-YFP. DS9 and DS10 stably propagate distinct tau aggregate conformations and exhibit unique inclusion morphology, biochemistry, and phenotypes in culture and in vivo [16, 22]. DS1 is a negative control cell line that expresses only monomeric tauRD(P301L/V337M)-YFP.

### Transgenic animals

PS19 transgenic mice that express 1N4R P301S human tau under the murine prion promoter [27] were purchased from Jackson Laboratory. They were maintained on a B6C3 background, and raised with wild-type (WT) littermates. Mice were provided food and water *ad libitum,* and housed under a 12-hour light/dark cycle. All animal maintenance and experiments adhered to the animal care and use protocols of the University of Texas Southwestern Medical Center, and Washington University in St. Louis.

### Human tissue

The autopsy brains used for this study were obtained from five individuals (1 female, 4 males) in compliance with Ulm University ethics committee guidelines as well as German federal and state law governing human tissue usage. Informed written permission was obtained from all patients and/or their next of kin. All cases were neuropathologically staged according to published protocols [3–6, 15].

### Isolation of mouse brain

Animals were anesthetized with isoflurane and perfused with chilled PBS with 0.03% heparin. Whole-brains were drop-fixed in 4% paraformaldehyde (PFA) in PBS overnight at 4 °C. For time course samples, brains were first bisected; the left hemispheres were drop-fixed in 4% PFA, while right hemispheres were stored as fresh frozen tissue at−80 °C until use.

### Immunohistochemistry of mouse tissue

A freezing microtome was used to collect 50 μm free-floating coronal sections from fixed mouse brains or 50 μm sections from fresh frozen tissue. For immunohistochemistry (IHC) analysis of tau pathology in mice, sections were incubated with biotinylated AT8 antibody (1:500, Thermo Fisher Scientific) overnight at 4 °C. Sections were washed and incubated with VECTASTAIN Elite ABC Kit (Vector Labs) prepared in TBS for 30 minutes at room temperature. Samples were developed with 3’3-diaminobenzidine using the DAB Peroxidase Substrate Kit with optional nickel enhancement (Vector Labs). Sections were imaged using an Olympus Nanozoomer 2.0-HT (Hamamatsu). Tau pathology was assessed using ImageJ as previously described [13, 26]. Briefly, the regions of interest (entorhinal cortex/amgdala, dentate gyrus) were outlined, and tau AT8 pathology was analyzed by brightness thresholding. The area covered with AT8 staining was reported as a percentage of total area analyzed.

### Isolation of brain regions from mouse and human brain slices

Free-floating mouse brain regions were washed in 1x TBS (50 mM Tris-Cl, pH 7.5 150 mM NaCl) and placed under a dissection microscope. Single brain regions were collected with a 1 mm punch biopsy tool, and placed into 40uL per punch of 1x TBS in PCR tubes. To avoid cross-contamination of seeding activity, only one punch biopsy tool was used for each brain, and then discarded. Human brain tissue blocks fixed in 4% formaldehyde were embedded in polyethylene glycol (PEG 1000, Merck) and sectioned coronally at 100 μm, as described elsewhere [3, 25]. Tissue sections with the human transentorhinal cortex, entorhinal cortex, and CA1–4 with AT8-positive tau pathology and from CA1/3 of the mouse hippocampus (2.5 mm x 2.5 mm) were collected and placed into 1 x TBS for homogenization. Final concentration of mouse samples was 1 mm^3^ of tissue per 1 mL of TBS, while human samples were homogenized in 100 μL and then diluted to 10 mm^3^ per 1 mL of TBS (v/v).

### Paraffin embedding, sectioning, and tissue preparation

Paraffin sections from mice were collected as 10 x 5 μm left hemisphere coronal brain slices and stored in 100% ethanol until use. Excess paraffin was removed by performing 2 x 100% ethanol washes at 60 °C.

### Tissue homogenization

Mouse samples were sonicated in a water bath in PCR tubes for 30 minutes under 50% power at 4 °C (Qsonica Q700 power supply, 431MPX microplate horn, with chiller). For fresh frozen tissue homogenization, right hemisphere slices from aged mice were collected by freezing microtome as described above, and placed into 1 x TBS with protease inhibitors. These samples were kept on ice, and sonicated immediately after collection in parallel with fixed tissue slices taken from the left hemisphere. These samples were immediately transduced into biosensor cells as described below. Human tissue samples were sonicated under identical conditions for 60 minutes.

### Transduction into biosensor cell lines

Biosensor cells were plated at 25,000 cells per well in 96-well plates. After 18 hours, cells were transduced with mouse or human homogenates as previously described [13]. Tissue from the mouse aging study and human samples were used at stock concentrations prepared as described above, while samples from the spreading paradigm were diluted 1:5 in TBS. Tissue samples were added to Opti-MEM (Thermo Fisher Scientific) and incubated for 5 minutes (5 μL of mouse tissue lysate with 5 μL of Opti-MEM, or 3.3 μL human with 6.7 μL of Opti-MEM per well). Lipofectamine was incubated with Opti-MEM (1.25 uL Lipofectamine with 8.75 μL Opti-MEM per well) for five minutes. Lipofectamine complexes were then mixed with samples and incubated for 20 minutes prior to addition to biosensor cells.

Mouse tissue samples were assayed in triplicate, and human tissue samples were added in sextuplicate. Cells were kept at 37 °C in a humidified incubator for 24 hours for experiments with cell lysate or transgenic mouse brain homogenate, and for 48 hours for all human tissue experiments described here. Cells were subsequently collected and prepared for flow cytometry analysis.

### Inoculation of strains into transgenic mice

DS1, 9, or 10 cell lines were trypsinized from 3 x 10 cm dishes, pelleted, and washed with 1 x PBS. Cell pellets were frozen at−80 °C until use. Cell lines were frozen on ice and resupsended in 1 x PBS with cOmplete mini protease inhibitor tablet (Roche). Cells were sonicated at 4 °C with an Omni-Ruptor 250 probe sonicator at 30% power for 1-second pulses x 30 cycles. Cell lysate was clarified with a 1000 x g spin and the supernatant was stored at−80 °C until use.

PS19 mice that underwent stereotaxic inoculations were anesthetized with isoflurane. A regulated heating pad was used to maintain core body temperature throughout the procedure. Animals were inoculated in the left hippocampus with 20 μg of cell lysate (from Bregma: x =−2.0, y =−2.5, Z =−1.8), and kept for 3, 6, or 12 weeks after injection.

### Generation of strains in secondary cells

Fixed hippocampal sections from P301S mice injected with DS9 or DS10 and incubated for 12 weeks were collected and sonicated as above. LM1 cells were plated at 8000 cells per well of a 96-well plate and allowed to grow overnight. Wells were subsequently treated with 5uL of pooled, fixed tissue hippocampal sections from DS9 and DS10 mice (*n* = 3–4 mice per condition) and incubated for 48 hours. Cells were re-plated into a 12-well dish, and grown for an additional two days. Single cells were sorted by fluorescence activated cell sorting (FACS) into five 96-well plates per condition using the Beckman Coulter MoFlo at the Siteman Flow Cytometry core facility at the Washington University in St. Louis. Cells were observed for individual colony growth for 10 days. Single cell colonies that contained aggregates were isolated as monoclonal secondary cell lines and grown to confluency in 10 cm dishes. Cell pellets were collected for biochemistry, plated for confocal microscopy, and subsequently frozen in 90% FBS/10% DMSO for long-term storage.

### Immunocytochemistry of secondary cell lines

Secondary cell lines were plated at low confluency on glass cover slips (0.09 to 0.12 mm thickness; Carolina Biologicals) and allowed to grow for 72 hours. Cells were fixed with 4% PFA, permeabilized with 0.25% Triton X-100 and DAPI stained. Cells were imaged with a Zeiss LSM780 inverted confocal microscope. For secondary cell line seeding assays, protein content was normalized by a Bradford assay to 0.5 μg/μL, and 2 μg of cell lysate was transduced per well in triplicate. Transduction was performed as described above.

### Flow cytometry and analysis of seeding activity

Biosensor cell lines were harvested with 0.05% trypsin, and quenched with media (DMEM + 50% FBS, 1% Pen/Strep, 1% Glutamax). Cells were centrifuged at 500 x g and resuspended in 4% PFA in 1x PBS. Cells were subsequently centrifuged at 500 x g, resuspended in flow buffer (HBSS + 1% FBS + 1 mM EDTA), and stored for less than 24 hours prior to performing flow cytometry. All flow cytometry for biosensor cells treated with mouse and human-derived tissue was performed using a Miltenyi VYB flow cytometer. Flow cytometry data was analyzed as previously described [10]. Integrated FRET density was calculated as integrated FRET density (IFD) = (percentage of FRET-positive cells)*(median fluorescence intensity). IFD was normalized to negative control samples.

### Statistical analysis

All statistical analysis was performed using GraphPad Prism. Tau AT8 and seeding time course data were standardized from 0 to 100%, and modeled using nonlinear regression analysis as a log(agonist) vs. normalized response (variable slope). S_10_ and S_50_ refer to the log(EC_10_) and log(EC_50_) calculated from these curves. Unless explicitly stated, all statistical analyses used one-way analysis of variance with Bonferroni’s multiple comparison test.

## Results

### Fixed tissue retains tau seeding activity

Formaldehyde-fixed tissue samples are a stable resource to assess tau pathology by microscopy, but are not suitable for classical biochemistry. Nonetheless, fixed tissue from Creutzfeld-Jakob disease (CJD) patients retains prion infectivity and strain properties [20]. Moreover, PrP aggregates from fixed tissue will amplify natively folded PrP in the real time quaking-induced conversion assay (RT-QuIC) [14]. The RT-QuIC technique relies upon seeded aggregation of recombinant PrP by pathological PrP present in samples.

Fixed tissue that contains Aβ pathology also retains its seeding activity and conformation upon inoculation into transgenic mice [9]. Similarly, fixed samples with α-synuclein pathology can induce synucleinopathy after inoculation into transgenic mice that express human forms of α-synuclein [24]. Thus, several aggregation-prone proteins implicated in neurodegenerative diseases retain seeding activity after fixation.

**Fig. 1:**
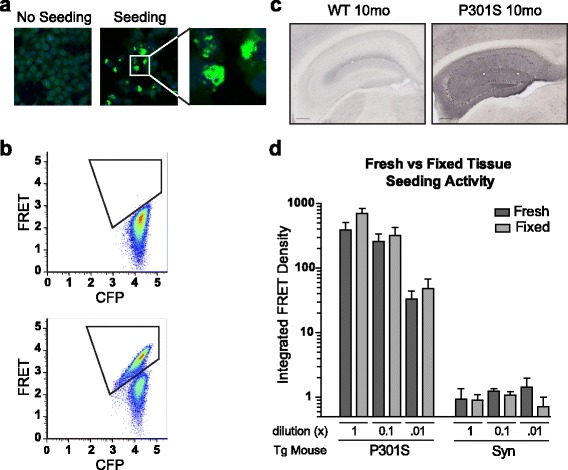
Fixed and fresh frozen tissue exhibit similar levels of seeding activity. **a** Transduction of aggregated tau into biosensor cells induces aggregation of endogenously expressed tauRD(P301S)-YFP fusion proteins. **b** Flow cytometry of tau biosensor cells can detects tau seeding based on FRET from aggregation of tauRD(P301S)-CFP and tauRD(P301S)-YFP fusion proteins. Note the population of cells that shift to the FRET-positive gate. **c** AT8 phospho-tau pathology is apparent in aged PS19 mice, whereas WT mice show no tau pathology. **d** Tau seeding activity is similar between fixed and fresh frozen tissue sections collected from aged PS19 mice. No significant difference was detected between fixed and fresh tissue at each concentration tested. Seeding is not detected in aged mice that express α-synuclein (A53T) [12]. Integrated FRET density is calculated as (Percent FRET-positive cells)*(median fluorescence intensity of positive cells). This value is normalized to a negative control sample. See Additional file 1: Figure S1a for an additional comparison of fixed versus fresh frozen tissue seeding, and Additional file 1: Figure S1b for seeding of fixed tissue embedded in PEG or paraffin. Error bars = S.E.M

We used the quantitative cell-based seeding assay to test whether tau protein also retains its seeding properties after fixation [10, 13]. This assay utilizes HEK cells that stably express tau four repeat domain (RD) with a P301S mutation fused to cyan or yellow fluorescent proteins (termed tau-CFP/YFP for brevity). Transduction of material that contains tau prion seeds into biosensor cells triggers aggregation of tau-CFP/YFP (Fig. 1a). This brings CFP and YFP into close association and results in fluorescence resonance energy transfer (FRET) that we quantify by flow cytometry (Fig. 1b) [10, 13].

Previous work has shown that fixed tissue samples with Aβ pathology can be lysed using a Precellys 24-Dual homogenizer [9]. However, this method yielded approximately 50% reduction in the level of seeding observed in fresh-frozen tissue. Sonication-based homogenization techniques have been utilized to detect minute amounts of misfolded PrP [21]. Thus, we adopted an extended water-bath sonication protocol to homogenize fixed tissue samples.

PS19 mice that express 1N4R tau(P301S) under the prion promoter develop progressive pathology over ~1 year [27]. Tau seeding activity was compared in fresh frozen versus fixed tissue samples from mice at 10 months, when these animals show robust tau pathology (Fig. 1c). In parallel, fixed tissue slices were collected and homogenized from the left hemisphere and equivalent fresh frozen tissue slices from the right hemisphere. Fixed and fresh frozen tissue similarly seeded aggregation after transduction into biosensor cells (Fig. 1d). In contrast, fixed and fresh frozen slices from negative control transgenic mice that express α-synuclein (A53T) under the prion promoter [12] did not induce aggregation in this biosensor line (Fig. 1d) nor did tissue from a wild-type (WT) mouse (Additional file 1: Figure S1a).

To verify that seeding can be detected in fixed tissue processed with other embedding protocols, we tested brains from aged P301S mice embedded in paraffin or PEG. Paraffin-embedded sections required 100% ethanol washes at 60 °C to remove excess wax, while PEG was readily removed with aqueous buffer. We detected seeding activity in samples embedded in both PEG and paraffin (Additional file 1: Figure S1b).

### Quantification of seeding activity in fixed tissue from PS19 mice

**Fig. 2:**
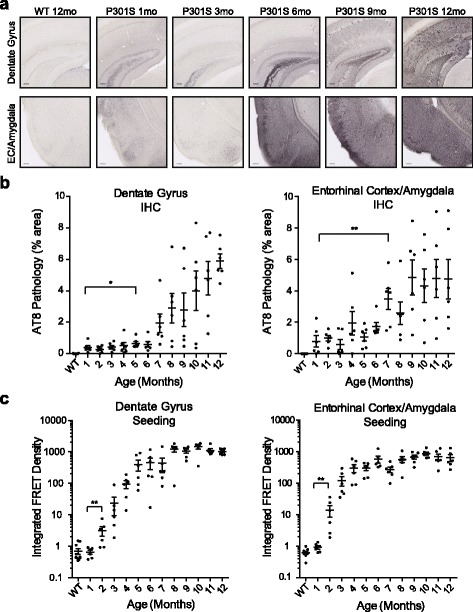
Seeding activity increases with age and anticipates AT8 pathology in PS19 mice. **a** AT8 staining of phospho-tau pathology in the dentate gyrus (DG) and entorhinal cortex/amygdala (EC/A) of a 12 month old WT mouse, and in PS19 mice at 1, 3, 6, 9, and 12 months of age. AT8 staining increases with age in PS19 mice. See Additional file 2: Figure S2a for representative images of whole-brain slices from 12-month-old WT and PS19 mice. **b** AT8 tau pathology was quantified in the DG and EC/A of WT and PS19 mice. WT mice were collected from various ages. Six PS19 mice at each age were assessed for tau pathology. Threshold analysis was performed to quantify the percentage of area occupied by AT8 staining in the region of interest. DG AT8 pathology did not rise above baseline staining in 1 month mice until 5 months of age. EC/A AT8 pathology did not show significant increases until 7 months of age. See Additional file 2: Figure S2b for nonlinear regression model of time-course AT8 staining data, and Table 1 for details regarding mice used in this study. **c.** Seeding activity was assessed from adjacent free-floating brain slices of WT and PS19 mice. 1 mm punch biopsies from the DG and EC/A were homogenized and transduced into biosensor cells. Tau seeding activity increased above baseline by 2 months of age for both the DG and EC/A. See Additional file 2: Figure S2b for nonlinear regression model of tau seeding activity time course data, and Additional file 2: Figure S2c for a direct comparison of seeding activity versus AT8 tau pathology. Error bars = S.E.M; * = *p* < .05; ** = *p* < .01

IHC is typically used to assess phosphorylated tau pathology in tauopathy autopsy samples [5, 17]. To directly compare seeding activity to IHC, brain slices were taken from PS19 mice at 1–12 months of age. Aged WT cage-mates served as negative controls. We assessed tau AT8 histopathology in the dentate gyrus (DG) and entorhinal cortex/amygdala (EC/A). In PS19 mice, we observed increasing levels of AT8 tau pathology over time, whereas in WT mice no phospho-tau pathology was detected even at 12 months of age (Fig. 2a, Additional file 2: Figure S2a, Table 1).

**Table 1:** Aging PS19 mouse study

Age at Collection	*N* (Total)	Male	Female
P301S Mice			
1 month	6	3	3
2 month	6	3	3
3 month	6	3	3
4 month	6	3	3
5 month	6	3	3
6 month	6	3	3
7 month	6	3	3
8 month	6	3	3
9 month	6	3	3
10 month	6	3	3
11 month	6	3	3
12 month	6	3	3
WT Mice			
1 month	4	3	1
2 month	1	0	1
5 month	1	0	1
6 month	1	1	0
9 month	1	1	0
10 month	1	0	1
12 month	1	0	1

AT8 pathology in the DG of PS19 mice did not exceed low baseline levels until 5 months of age (Fig. 2b). The EC/A exhibited a more apparent, but highly variable AT8 signal from early ages, but this did not rise above the baseline until 7 months (Fig. 2b). The low dynamic range of AT8 staining in this mouse model hinders quantitative assessment of pathology using this metric.

Next, we measured seeding activity in 1 mm punch biopsies from fixed tissue slices immediately adjacent (50 μm) to those stained for AT8 pathology. We homogenized biopsies from the DG and EC/A, transduced them into the biosensor cell line, and assessed them after 24 hours (Fig. 2c). In PS19 mice, the DG and EC/A exhibited higher seeding activity at 2 vs. 1 month. As reported earlier with unfixed samples [13], we observed a 1000-fold dynamic range between negative control WT brains and aged PS19 mouse samples, which facilitated this quantitative analysis even among samples with relatively variable patterns of histopathology.

To determine the rate at which seeding activity and AT8 pathology develop in the PS19 mouse line, we standardized data from both metrics from 0 to 100% and performed nonlinear regression analyses. Seeding reached 10% (S_10_) and 50% (S_50_) of maximum signal earlier than AT8 pathology both in the DG and EC/A (Additional file 2: Figure S2b). We also plotted seeding activity against the AT8 pathology from individual mice. Seeding activity increased earlier than AT8 pathology in individual mice. We observed robust phospho-tau pathology above 6 months of age in the DG and above 3 months in the EC/A, whereas seeding scored positive much earlier (Additional file 2: Figure S2c). We conclude that tau seeding activity measured in fixed tissue is a robust and highly sensitive metric for tau pathology, and anticipates AT8 staining in this mouse model.

### Quantification of spreading tau pathology

**Fig. 3:**
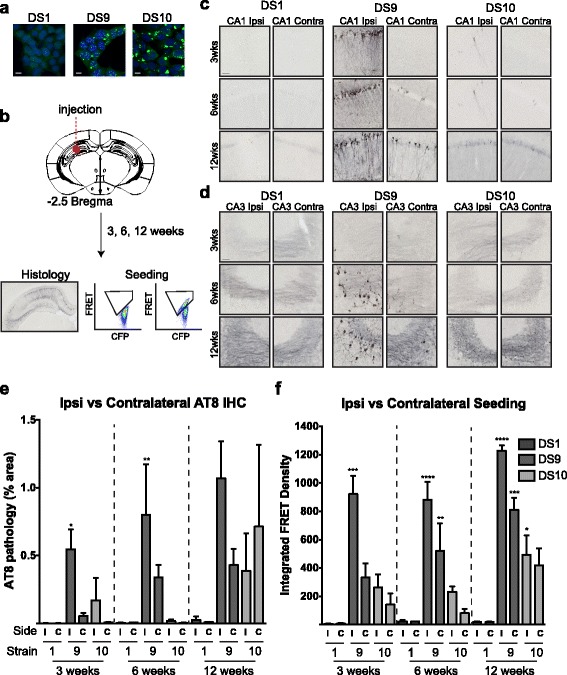
Seeding activity detects spread of tau pathology. **a** Tau strains DS1, 9, and 10 have different inclusion morphologies. DS1 does not contain aggregated tau. DS9 cells feature nuclear speckles, while DS10 cells have a large juxtanuclear aggregate and no nuclear speckles. **b** Cell lysate from DS1, 9, or 10 was inoculated into the hippocampi of young PS19 mice. At 3, 6, or 12 weeks, brains were collected for tau histopathology and seeding analysis. **c** Inoculation of DS1 did not induce AT8 pathology. Mice inoculated with DS9 developed NFT-like AT8 pathology in CA1 of the ipsilateral hippocampus by three weeks. This pathology spread to the contralateral hippocampus by 6 weeks. DS10 produced limited tau pathology in this region. **d** DS9 inoculation induced neurofibrillary tangle-like pathology in CA3 of the ipsilateral hippocampus, and limited pathology in the contralateral hippocampus by six weeks. DS10 inoculation primarily induced mossy fiber AT8 pathology that progressed over time, and spread to the contralateral hippocampus by 12 weeks. **e** The percentage of the hippocampus covered with AT8 tau pathology was assessed in mice inoculated with DS1, 9, and 10 at each time point. Tau AT8 pathology and spread was apparent in DS9 inoculated mice. However, DS10 mossy fiber pathology was difficult to detect with this technique and showed variable pathology among animals (**f**). Tau seeding activity was detected in ipsilateral and contralateral hippocampi of DS9 and DS10 inoculated mice at 3 weeks. Seeding activity increased by 12 weeks, suggesting tau pathology continues to develop over time. ANOVA analysis was performed by comparing samples within each time point to DS1 inoculated controls. Error bars = S.E.M, * = *p* < 0.05, ** = *p* < 0.01, *** = *p* < 0.001, **** = *p* < 0.0001

Tau pathology progressively accumulates along neuronal networks in AD patient brains [4]. We have developed an in vivo model of tau propagation through inoculation of distinct tau strains (DS1 (monomer control), DS9, DS10) derived from stable cells into the hippocampus of PS19 mice (Fig. 3a) [22]. To compare the utility of the fixed tissue seeding assay with IHC, we collected brain samples at 3, 6, or 12 weeks after injection and assessed pathology by AT8 stain or seeding activity (Fig. 3b, Table 2). As previously described, DS9 produced neurofibrillary tangle (NFT)-like pathology in CA1 and CA3, while DS10 induced mossy-fiber pathology (Fig. 3c,d). By 6 weeks, we observed spreading of phospho-tau pathology into CA1 of the contralateral hippocampus of DS9-inoculated mice. At this time point in mice inoculated with DS10, we observed mossy fiber pathology in the contralateral hippocampus, but limited CA1 pathology (Fig. 3c,d).

**Table 2:** Inoculation model of spread of tau pathology

Time Course	*N* (Total)	Male	Female	Average Age at Surgery (Days)	Average Age at Collection (Days)
3 weeks					
DS1	2	0	2	94	115
DS9	4	0	4	90	111
DS10	4	0	4	91	112
6 weeks					
DS1	3	0	3	77	119
DS9	4	1	3	80	122
DS10	4	0	4	80	122
12 weeks					
DS1	2	0	2	71	155
DS9	4	2	2	70	154
DS10	3	1	2	71	155

We quantified tau histopathology by determining the percentage area covered by AT8 staining in the ipsilateral and contralateral hippocampi of inoculated mice. DS1-injected mice exhibited limited AT8 pathology at this time point. DS9 and DS10 inoculates induced increasing levels of pathology in the hippocampus, but threshold analysis of AT8 staining was difficult in DS10 mice owing to the subtle mossy fiber phenotype (Fig. 3e).

To assess tau seeding activity, we dissected and homogenized ipsilateral and contralateral hippocampi from fixed free-floating 50 μm brain sections of inoculated mice. We transduced hippocampal tissue into biosensor cells, and quantified seeding activity by flow cytometry after 24 hours. Mice inoculated with DS1 exhibited a minimal signal possibly due to the endogenous seeding activity present at this age (Fig. 2c, Fig. 3f). Mice inoculated with DS9 and DS10 exhibited robust seeding activity that increased over time in the ipsilateral hippocampus. We also detected clear seeding activity in the contralateral hippocampus 3 weeks after injection with DS9 or DS10, in contrast to the AT8 staining. We conclude that seeding activity in fixed tissue anticipates the spread of tau pathology and is robust prior to AT8 pathology detectable by immunohistochemistry.

### Fixed tissue retains strain-specific conformations

After fixation, PrP and Aβ retain their ability to serve as distinct conformational templates [9, 19]. To test whether tau strains similarly retain their conformations after fixation, we homogenized fixed hippocampi from mice inoculated with DS9 or DS10 12 weeks post-injection. We transduced hippocampal lysate into the LM1 biosensor cell line. After four days, we used FACS to isolate aggregate-containing monoclonal cell lines in 96-well plates.

**Fig. 4:**
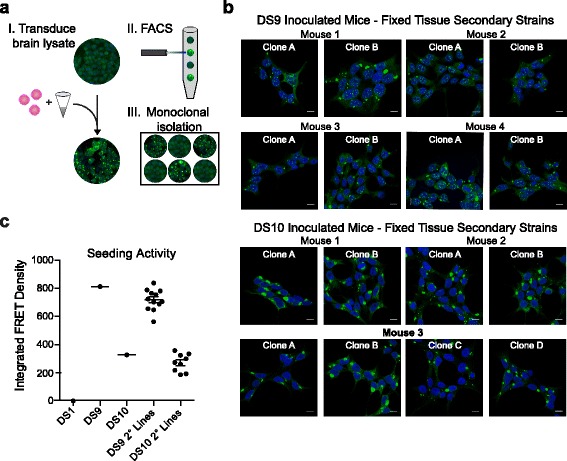
Strain-specific properties are retained after fixation. **a** Fixed hippocampi were isolated from PS19 mice at 12 weeks post injection with DS9 or 10. This tissue was homogenized and transduced into the original LM1 cell line. Fluorescence-activated cell sorting was used to isolate monoclonal cells into 96-well plates. Cells that stably propagated aggregates were amplified and characterized. **b** Confocal images of representative secondary cell strains derived from mice inoculated with DS9 or 10. Secondary strains displayed the same inclusion morphology as the original inoculum (nuclear speckles or a large juxtanuclear aggregate). See Fig. 3a for images of original strain morphology. **c.** Seeding activity was assessed for DS9 and 10, as well as secondary cell lines. Cell lysate from each line was transduced into biosensor cells and assessed for tau seeding activity after 24 hours (2 μg per well). Secondary strains showed similar seeding activity to the original inoculum. See Table 2 for additional information regarding the mice used in this study

We examined the wells for cell colony growth at 7–10 days post-plating; 8% of DS9 derived wells and 5% of DS10 derived wells contained aggregate-positive cell colonies at 7–10 days. As observed previously, the initial passage of isolated monoclonal secondary cell lines is toxic. Thus, 24 DS9-derived secondary lines and 9 DS10-derived lines were isolated. Three secondary cell lines from each DS9 inoculated mouse and all DS10 derived cell lines (2–4 lines per mouse) were analyzed for morphology and/or seeding activity (Fig. 4a).

Secondary cell lines derived from mice inoculated with DS9 and DS10 displayed cellular morphologies identical to the original strains as seen in Fig. 3a (e.g., nuclear speckles versus no nuclear speckles and a large juxtanuclear aggregate) (Fig. 4b). This is consistent with previous results from fresh frozen tissue, which demonstrated that tau strains stably template onto human tau expressed in PS19 mice and then back into LM1 cells [22]. Secondary cell lines derived from fixed tissue showed similar seeding activity to the original DS9 and 10 strains (Fig. 4c). Thus, tau strains retain their conformation and strain-specific properties in young and aged mice even after fixation.

### Quantification of seeding activity in fixed tissue from the human brain

**Fig. 5:**
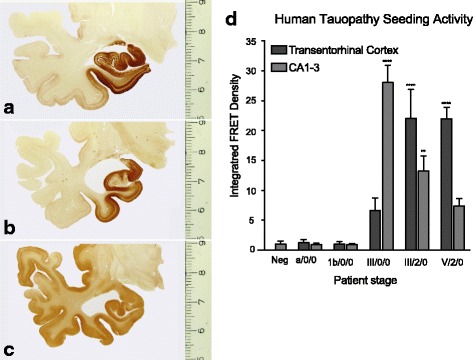
Fixed human brain samples with tau pathology exhibit seeding activity. AT8-immunostained (hyperphosphorylated tau, DAB) 100 μm sections from cases 3 (**a**) and 4 (**b**) in Table 3. In NFT stage III, the tau pathology in the hippocampal formation increases. **a**,**b**. The entorhinal layers pre-α and, in addition, pri-α become heavily involved. Tau pathology extends through the transentorhinal region into the adjoining high order sensory association areas of the temporal neocortex but not yet into the superior temporal gyrus. **c** The NFT in the late stage V case shown here is not identical to case 5 in Table 1 but is from another Alzheimer’s disease patient in her eighties. During NFT stage V, tau lesions develop in the superior temporal gyrus and progress into first order sensory association and premotor areas of the neocortex. **d** Fixed tissue was isolated from the transentorhinal cortex and the hippocampus (CA1/3) of 100 μm human brain sections that were blinded prior to collection (Table 3). Samples were homogenized and transduced into tau biosensor cells. The integrated FRET density was normalized to a negative control treated only with Lipofectamine. Error bars = S.E.M, ** = *p* < 0.01, **** = *p* < 0.0001

To test the seeding assay in formaldehyde-fixed human tissue samples, we examined the transentorhinal cortex and Ammon’s horn (CA1/3) of five individuals with different stages of tau pathology (Table 3) [4]. While we observed robust seeding from PS19 mice, they express an aggregation-prone form of tau at approximately 5x the level of endogenous tau expression in humans [27]. To increase the sensitivity of the assay, human samples were added to biosensor cells at 10x concentration and the incubation period was extended to 48 hours. Subjects with no tau pathology or with very subtle pretangle pathology (stages a/1b) did not display detectable seeding activity in either region. In contrast, tissue from individuals with NFT stages III and V tau pathology contained seeding activity in both the transentorhinal cortex and Ammon’s horn (Fig. 5; Table 3). Thus, tau seeding activity can be quantified from fixed mouse and archival human tissue alike.

**Table 3:** Human tauopathy and control subjects

Patient Case	NFT/Aβ/α-synuclein Stage	M/F	Age	Year of formalin fixation	Notes
1	a/0/0	M	31	1989	Control
2	1b/0/0	M	42	1989	Control
3	III/0/0	F	90	1992	No clinical AD diagnosis
4	III/2/0	M	65	1989	No clinical AD diagnosis
5	V/3/0	M	88	1990	Clinical AD

## Discussion

Propagation of tau aggregation along neuronal networks may mediate the progressive accumulation of pathology observed in tauopathy patients. To measure tau seeding activity in well-characterized human brains, it will be necessary to analyze formaldehyde-fixed tissues. We now present a method for extracting tau seeding activity from miniscule amounts of fixed tissue (approximately .04 mm^3^) to permit direct comparison with tissues stained by IHC.

We first tested this method in PS19 mice that overexpress full-length human tau (1 N,4R) containing the P301S mutation. We drop-fixed brain samples that had been embedded either in paraffin or PEG and sectioned them coronally for microscopy. We analyzed adjacent 50 μm sections using standard IHC to detect phospho-tau or 1 mm circular punch biopsies of tissue for seeding assays. We homogenized punch biopsies by water-bath sonication in closed tubes, and assayed them in a cellular FRET bioassay system as described previously [10, 13].

Tau seeding activity tracked the development of pathology more efficiently than IHC, with a lower degree of inter-animal variation, and a higher dynamic range. This was perfectly comparable to previously obtained results using fresh frozen tissue [13]. In addition, we detected seeding activity relatively early in the course of disease (1–2 months) and it steadily increased over time. Next, we tested brain tissues from animals previously inoculated with two distinct tau prion strains. We recovered these strains from fixed mouse brain tissue as accurately as we had previously from fresh frozen tissue. Finally, we tested the extraction method in fixed human brain tissue with documented AT8-positive tau pathology, including AD, and readily detected tau seeding activity in cases archived for up to 27 years in formaldehyde.

### Seeding activity

Our laboratory previously detected tau seeding activity in fresh frozen brain tissue from mouse tauopathy models and human AD cases[11, 13]. However, fresh frozen samples are much more difficult to obtain than fixed tissue sections, must be carefully stored at−80 °C, and are very challenging to dissect precisely to isolate specific brain regions. The assay described here accurately quantifies tau seeding from fixed tissue sections over three log orders of signal. Remarkably, in a mouse model from which we sampled tissue at different time points, fixed tissue seeding proved comparable to seeding activity detected in fresh frozen tissue. Thus, we expect that this assay will enable assessment of tau seeding activity in a range of fixed tissues at a similar level of sensitivity to fresh frozen samples.

Moreover, we detected seeding activity in a small sample of human tauopathy cases that were collected and stored in formalin for over 20 years prior to this study. We observed lower seeding activity in these human samples than in PS19 mice, probably because of the overexpression of an aggregation-prone form of tau in this mouse model. However, the length of fixation may affect the level of seeding observed in samples. Further, differences in seeding activity observed between patients at Braak stage III and V likely reflect differences in the level of tau aggregate burden between these patients, cell loss, or ghost-tangle formation at later disease stages. Given the early detection of seeding activity relative to AT8 staining in PS19 mice, we anticipate that this assay could represent a more sensitive metric of tau pathology. Additional studies in a large number of well-characterized human tissue samples will help address these important questions, and provide additional insight into the progression of seeding activity in human tauopathies.

Earlier work described a dose-dependent increase in tau seeding activity in the PS19 mouse tauopathy model [13]. However, the regional specificity possible with fresh frozen tissue was limited to gross dissection. We now have reliably isolated and characterized punch biopsies as small as 1 mm diameter x 50 μm (or ~ .04 mm^3^). When we quantified the level of seeding activity at increasing ages vs. the tau pathology observed in adjacent tissue slices using anti-tau AT8 staining, we easily detected tau seeding activity, even in fixed tissue sections with a minimal AT8 signal. For example, when PS19 mice were inoculated with tau strains, we induced strong AT8 pathology with DS9, whereas DS10 produced a weak signal. In both cases, the pathology spread from the site of inoculation to connected regions, as described elsewhere [22]. The fixed tissue seeding assay more readily detected the spread of tau pathology in this propagation model. Furthermore, we readily detected seeding activity in DS10 inoculated mice despite the relatively subtle AT8 staining phenotype induced by this strain (mossy fiber dots). Consequently seeding activity can serve as an important measure of tau pathology when routine AT8 staining reports otherwise minimal pathology. The combination of precise quantification of seeding activity with the ability to sample brain tissue to 1 mm resolution indicates that this method could help define the seeding activity in human brain with remarkably high accuracy.

### Detection of tau strains in formaldehyde-fixed tissue

Prior experimental work indicates that distinct tau aggregate conformations may underlie different patterns of pathology, rates of progression, and disease phenotypes observed in distinct tauopathies [2, 7, 22]. Distinct tau strains are associated with different tauopathies [22], and inoculation of unique tau strains produces different patterns and tau pathology rates of progression [16]. We observed that fixed tissue from mice inoculated with DS9 and DS10 produced strain phenotypes identical to the original strains upon inoculation into LM1 biosensor cells. Thus, tau strains are stable upon fixation. We anticipate that formaldehyde-fixed tissues will serve as an invaluable resource to examine the role of strain composition in tauopathies.

Studies that use traditional IHC techniques to detect tau pathology have provided important insights into the progression and anatomy of macromolecular accumulations of tau assemblies. However, these methods cannot discriminate among distinct strains, nor can they detect submicroscopic tau assemblies. The present assay measures tau pathology based on seeding activity and is also sensitive to strain composition. We anticipate that punch biopsies taken from tissue sections will be useful to measure strain identity with high anatomical precision. By carefully comparing seeding activity and strain composition with standard neuropathology, it should be possible to add new dimensions to analyses of tissue samples from a range of neurodegenerative diseases. In turn, this will facilitate more widespread testing of the putative role of tau prion activity in human tauopathies.

## Additional files

Additional file 1: Figure S1. Fixed tissue reliably seeds tau aggregation. a. Comparison of fixed and fresh tissue seeding from aged PS19 mice. WT mouse tissue did not induce seeding. Seeding displays a dose-response, and no significant difference was detected at each concentration. b. Tau seeding was equivalently detected from aged PS19 brain tissue embedded in either paraffin or polyethylene glycol. Paraffin embedded tissue requires heated ethanol washes to remove excess wax prior to homogenization for robust seeding. A sham sample (Lipo) was used as a negative control. (PDF 97 kb)

Additional file 2: Figure S2. Seeding activity precedes AT8 pathology in PS19 mice. a. Representative images of 12 month WT and PS19 mouse hemi-brain slices stained with AT8. No AT8 staining was detected in WT mice, whereas PS19 mice exhibited robust phospho-tau pathology throughout the brain. b. Schematic of punch biopsy, transduction, and seeding assay workflow. c. Seeding and AT8 pathology time course data were modeled with nonlinear regression analysis using log (agonist) versus normalized response (variable slope). S_10_ and S_50_ refer to the time point at which seeding or AT8 pathology reaches 10% or 50% of maximal signal, and is represented in months. Seeding preceded AT8 pathology in both the DG and EC/A. d. Scatter plot analysis of tau seeding activity versus AT8 pathology for each animal. Seeding activity increases before robust AT8 pathology is observed in the DG and EC/A. (PDF 440 kb)
