# Supplementary figures and images for: Characterization of tau prion seeding activity and strains from formaldehyde-fixed tissue
Source: Acta Neuropathol Commun. 2017 Jun 7;5:41. doi: 10.1186/s40478-017-0442-8 (PMC5461712; doi:10.1186/s40478-017-0442-8)

**a**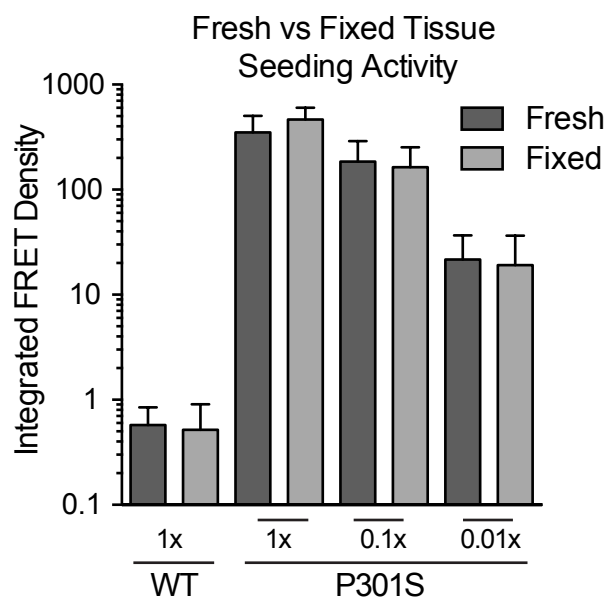**b**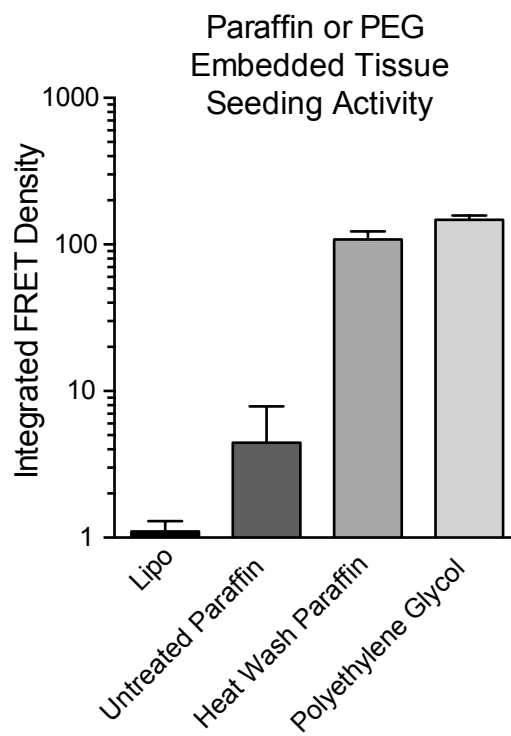

Supplement: Supplementary file 1 — Fixed tissue reliably seeds tau aggregation. a. Comparison of fixed and fresh tissue seeding from aged PS19 mice. WT mouse tissue did not induce seeding. Seeding displays a dose-response, and no significant difference was detected at each concentration. b. Tau seeding was equivalently detected from aged PS19 brain tissue embedded in either paraffin or polyethylene glycol. Paraffin embedded tissue requires heated ethanol washes to remove excess wax prior to homogenization for robust seeding. A sham sample (Lipo) was used as a negative control. (PDF 97 kb) [file 40478_2017_442_MOESM1_ESM.pdf]

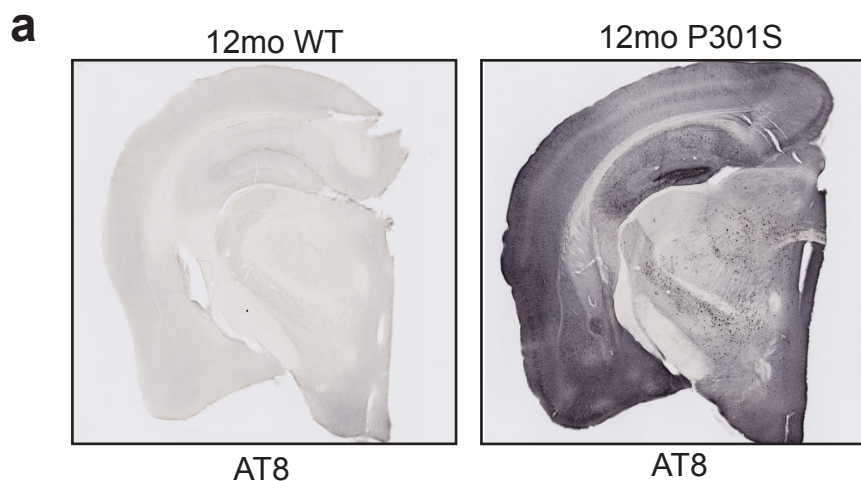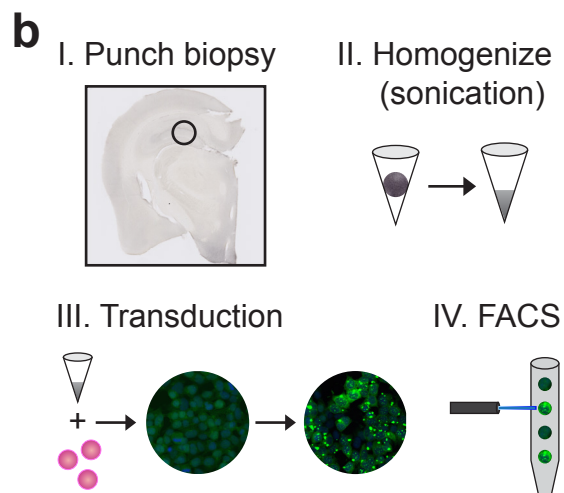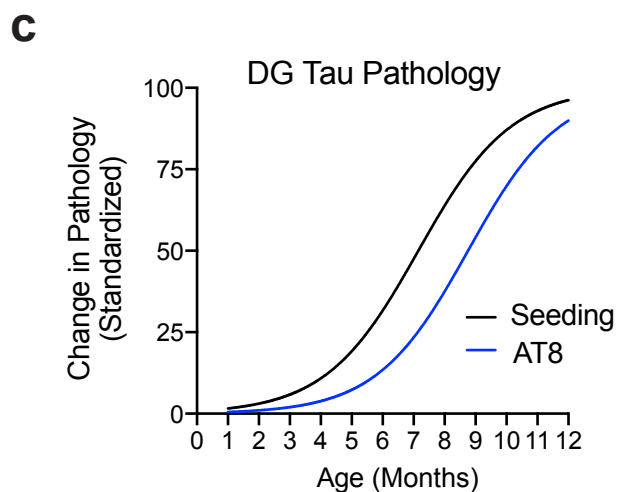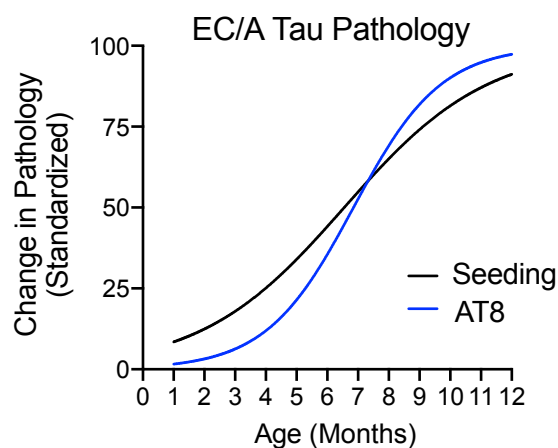

|          | Seeding  | AT8                  |
|----------|----------|----------------------|
| $S_{10}$ | 3.87 mos | 5.5 mos <sup>1</sup> |
| $S_{50}$ | 7.15 mos | 8.76 mos             |
| $R^2$    | 0.87     | 0.97                 |

|          | Seeding  | AT8      |
|----------|----------|----------|
| $S_{10}$ | 1.43 mos | 3.72 mos |
| $S_{50}$ | 6.55 mos | 6.85 mos |
| $R^2$    | 0.83     | 0.90     |

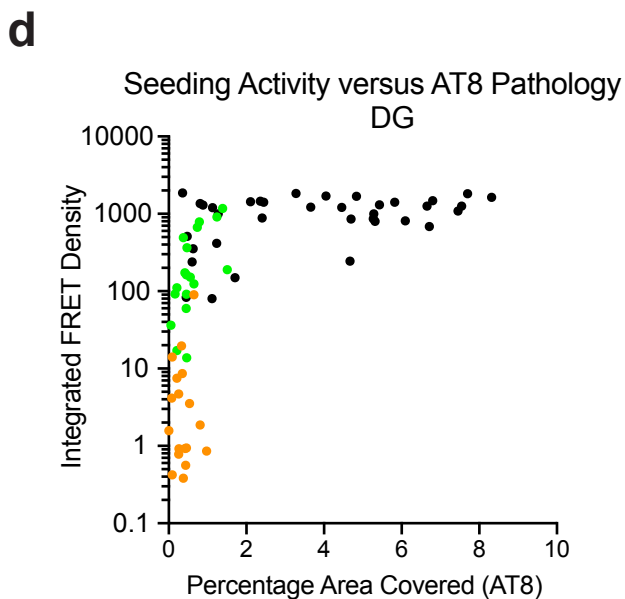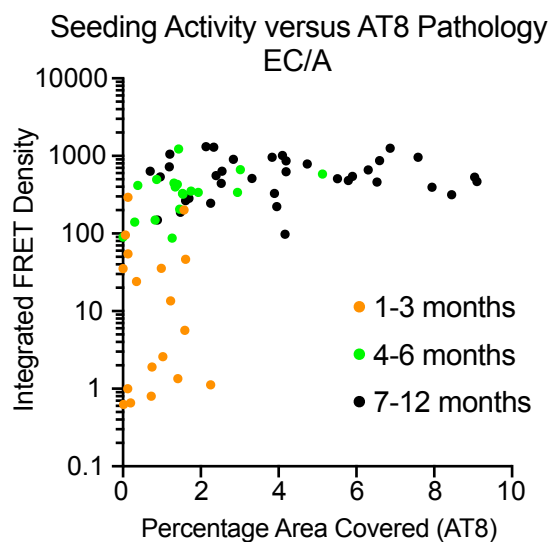

Supplement: Supplementary file 2 — Seeding activity precedes AT8 pathology in PS19 mice. a. Representative images of 12 month WT and PS19 mouse hemi-brain slices stained with AT8. No AT8 staining was detected in WT mice, whereas PS19 mice exhibited robust phospho-tau pathology throughout the brain. b. Schematic of punch biopsy, transduction, and seeding assay workflow. c. Seeding and AT8 pathology time course data were modeled with nonlinear regression analysis using log (agonist) versus normalized response (variable slope). S10 and S50 refer to the time point at which seeding or AT8 pathology reaches 10% or 50% of maximal signal, and is represented in months. Seeding preceded AT8 pathology in both the DG and EC/A. d. Scatter plot analysis of tau seeding activity versus AT8 pathology for each animal. Seeding activity increases before robust AT8 pathology is observed in the DG and EC/A. (PDF 440 kb) [file 40478_2017_442_MOESM2_ESM.pdf]
